# Supplementary material for: Early warning signals of the termination of the African Humid Period(s)
Source: Nat Commun. 2024 May 7;15:3697. doi: 10.1038/s41467-024-47921-1 (PMC11076281; doi:10.1038/s41467-024-47921-1)
Supplement: Supplementary file 1 — Supplementary Information [file 41467_2024_47921_MOESM1_ESM.pdf]

Supplementary information

## Early warning signals of the termination of the African Humid Period(s)

Martin H. Trauth[1]*, Asfawossen Asrat[2,3], Markus L. Fischer[1], Peter O. Hopcroft[4], Verena Foerster[5], Stefanie Kaboth-Bahr[6], Karin Kindermann[7], Henry F. Lamb[8,9], Norbert Marwan[10], Mark A. Maslin[11], Frank Schaebitz[5], Paul J. Valdes[12]

[1] University of Potsdam, Institute of Geosciences, Potsdam, Germany
[2] Botswana University of Science and Technology, Department of Mining and Geological Engineering, Palapye, Botswana
[3] Addis Ababa University, School of Earth Sciences, Addis Ababa, Ethiopia
[4] University of Birmingham, School of Geography, Earth & Environmental Sciences, Birmingham, United Kingdom
[5] University of Cologne, Institute of Geography Education, Cologne, Germany
[6] Free University Berlin, Institute of Geological Sciences, Berlin, Germany
[7] University of Cologne, Institute of Prehistoric Archaeology, Cologne, Germany
[8] Aberystwyth University, Department of Geography and Earth Sciences, Aberystwyth, UK
[9] Trinity College Dublin, Botany Department, School of Natural Sciences, Ireland
[10] Potsdam Institute for Climate Impact Research, Potsdam, Germany
[11] University College London, Geography Department, London, UK
[12] University of Bristol, Bristol Research Initiative for the Dynamic Global Environment, School of Geographical Sciences, Bristol, UK

*Corresponding Author: email trauth@uni-potsdam.de

## Supplementary Note: Characteristics of the climate tipping point and early warning signals in the sediment

The observation of a tipping of the climate at the end of the AHP associated with at least fourteen droughts, each 20–80 yrs long and recurring every 160±40 yrs and documented by 2–7 original data points (prior to interpolation), was discovered early in the potassium (K) record of short core CB01 but not studied in detail[1,2] (Suppl. Fig. 1, Suppl. Tab. 1). In core CB01, these droughts occur at composite depths between ~380–460 cm, with possibly two additional droughts above 380 cm (Suppl. Fig. 2). Between 460–480 cm, the K value is comparatively constant, while between 480–490 cm it is consistently lower (and more variable). The picture below 490 cm is inconsistent with

possible additional short droughts near 500 cm. Looking at data from all cores CB01–06 along the transect of short cores, the episodes with high K values, both the short ones above 460 cm and below 500 cm, but also the long one between 480–490 cm, can be observed not only in CB01, but with less distinct due to the generally lower quality of the records towards the center of the basin[3] (Suppl. Fig. 1).

To decipher the process of formation of the high-K episodes in the Chew Bahir record, it is first worth taking a look at the record of the other chemical elements or the optical appearance of core CB01 (Suppl. Fig. 2). Comparing the course of the K curve, in particular the appearance of high-K episodes with the coloring of the sediment core and the appearance of cracks, a weak correlation can be noticed. In fact, there are several light-colored layers and/or cracks that appear near–but not exactly at the location–of the K excursions, especially between 420–440 cm composite depth. The longer episode of elevated K values between 480–490 cm is not reflected in the sediment coloration in any way, nor do cracks occur in this area, which is why we will subject this section to a separate investigation (Suppl. Fig. 2).

A possible explanation for the weak but not exact spatial correlation could be related to the formation process of the K proxy as an indicator of an arid climate[4]. As earlier investigations suggest, the most likely process responsible for linking an arid climate and this proxy is a K fixation in smectites during illitization progressively enhanced by Al-to-Mg substitution in the octahedral layer due to resulting changes in the layer charge during dioctahedral to trioctahedral transition in clay minerals[4]. Thus, the K content in the Chew Bahir records can be largely attributed to a changing hydrochemistry that is controlled by fluctuations in the moisture influx, which in turn governs the precipitation/evaporation ratio of the closed paleo-lake[4]. In contrast, K (and other elements involved in feldspar weathering, such as Na and Ca) has traditionally been considered a function of weathering in the catchment and thus a humidity indicator, rather than an aridity indicator[6]. This process certainly plays a role, albeit a minor one, in the Chew Bahir, as shown by the characteristic shape of the K curve[4,5].

The light coloration of the sediment and the occurrence of cracks near the K maxima can be both explained by this process (Suppl. Fig. 2). The changes in the layer charge during dioctahedral to trioctahedral transition in clay minerals is perhaps aided by Fe reduction from $Fe^{3+}$ to $Fe^{2+}$ and hence a decolorization of the sediment[5]. Furthermore, the progressive smectite to illite transformation is accompanied by an increase in sediment density due to loss of OH molecules in the intermediate layers of the smectites, which gradually become illites. Smectites have densities

between 2–3 g/cm$^3$, depending on water content, while the mica-like illites have average densities of 2.6–2.9 g/cm$^3$. This 10–20% increase in density explains the appearance of shrinkage cracks in the sediment, which occur near, but for mechanical reasons not exactly at the position of the K maxima, and thus the abundance of illite relative to smectite. However, the spatial proximity, but not exact correspondence, of light-colored sediment layers and fractures is thus a consequence, rather than a cause, of K maxima.

For a deciphering the processes behind the K curve, especially the high-K interval between 480–490 cm, it is worth looking at the other chemical elements (Suppl. Fig. 2). A total of 61 chemical elements were measured in the µXRF scans. The variation of most elements is that of white noise or is very similar to it, either because the contents are below the detection limit or there is no or only a weak deterministic process which causes a variation different from noise (e.g., P, Pb, Pt, U, W, and Zr). For some elements, the signal-to-noise ratio is just sufficient to indicate the long-term wet-dry trend at the end of the African Humid Period (AHP), although here it cannot be ruled out that it is a dilution effect that causes this trend (e.g., Cl, S, Sr, Th, Ti, and Yb)[5,7,8].

Some elements show distinctly different trends from white noise, but these are complicated in the way that they do not show a simple wet-dry transition at the end of the AHP, nor do they show the precursor events mentioned above (Suppl. Fig. 2). This includes the Si-Al system, where Al shows a very weak trend towards higher values, while Si shows a step-like change from low to high values at the end of the AHP. Si also shows some rapid fluctuations, e.g., in the near the rapid K fluctuations at ~500 cm, the cause of which is unknown. Some of the high K events are associated with high Si values, while others are not. The variations in the Si-Al system are probably the results of several superimposed processes, such as the primary production of diatoms in the photic zone of the lake, influx of silicates into the lake, and early diagenetic processes in the sediment including the dissolution of diatom frustules and volcanic glass.

Another complicated system is the Fe-S system, complicated by the fact that the µXRF data do not distinguish between $Fe^{2+}$ to $Fe^{3+}$. In this system, as mentioned above, S shows a weak trend corresponding to the expected course of the wet-dry transition at the end of the AHP (Suppl. Fig. 2). Superimposed on this trend is a rapid variation that is not correlated with either the high K events or the coloration of the sediment and is therefore probably simply noise. The higher S values during the AHP could be due to higher contents of pyrite, which is presumably present in gypsum after its weathering. Here $Fe^{3+}$ converted to $Fe^{2+}$, which is accompanied by a green-gray to reddish-brown color change of the sediment. Fe shows a clear transition from low to high values at the end of the

AHP, in some cases a positive correlation with the K curve in the high K events, and increased values in the longer high K episode between 480–490 cm. There are also noticeable Fe fluctuations at around 500 cm, the cause of which is unknown.

The Ca-Sr system is also complicated and the result of overlapping processes that control the concentration of these elements with different sign. As said before, Ca, Na and K in the detritus delivered from the catchment area are, more or less, the result of feldspar weathering and hence a function of humidity rather than aridity in most sedimentary basins. After observing the dominant but opposite effect in the formation of the K proxy in the illitization of smectites, it is no surprise that Ca–and also Sr–also tends to indicate aridity, at least on long wavelengths. In contrast, during 20–80 yr droughts, indicated by the high K events, low Ca and Sr values tend to be indicated, but the spatial correlation in the core with K is not perfect. This could indicate that Ca and Sr are reflecting local occurrences of authigenic carbonates in the sediment, possibly also shells of organisms such as ostracods. Both Ca and Sr show a similar pattern with a rapid increase to higher values between 400–410 cm at the end of the AHP. In the high K events as well as in the variations between 480–490 cm and around 500 cm higher values are obtained, compared to other elements the correlation is quite good (Suppl. Fig. 2). Last but not least high Ca counts could also represent very dry conditions in the basin which are due to intensified evaporation processes.

The high K interval between 480–490 cm is difficult to explain, whether or not it is due to a longer episode of drier conditions or whether it reflects a rather local anomaly in the composition of the sediment (Suppl. Fig. 2). The latter could be indicated by significant increases in K but also in elements such as Cu, Fe, Rb, Ti, V, and Zn, for example, as a result of erosion of ore veins in the lake's catchment. Unfortunately, there is no detailed mapping of such veins, but their occurrence in the area of granitoid rock series would not be surprising[9]. In addition to the high K events, which likely indicate droughts, there are also at least seven low K events above 420 cm, namely at ~410 cm, ~400 cm, 395 cm, 385 cm, 375 cm, and 370 cm (Suppl. Fig. 2). These events are generally somewhat broader, i.e., longer on the time axis, than the high K events. In contrast to the low K phases of the section below 420 cm, which represent a kind of baseline of downward deflections in K, these are actually pronounced maxima and thus not simply an artifact of the high K events, but real wet episodes and hence they complement the high K events to a flicker of climate during the ~850 yrs long transition from wet to dry climate at the end of the AHP.

After this detailed comparison of the course of the K-curve with the sediment, we now examine the long core CHB14-2 for similar transitions and excursions in the K concentration. Indeed, different

types of transitions and variabilities are found in the ~620 kyr long record[2,7] (Suppl. Fig. 3). However, several transitions stand out, which are very similar to each other and all to the transition at the end of the AHP (Suppl. Fig. 3, Suppl. Tab. 2). Of these transitions, the younger ones (including the termination of the AHP) have a consistent duration of 3–6 kyrs, while the older ones are significant longer (up to 15.7 kyrs). Since the signal-to-noise ratio and the temporal resolution of the long core (~7–50 yrs) is slightly lower than that of the upper part of the short core studied here (~7–12 yrs) the examination of the K excursions, similar to the ones in the short core is difficult, but they are clearly visible and go in both directions (dry and wet) (Suppl. Fig. 3).

What is striking, however, and visible similarly to the short core at the termination of the AHP, is that the flickering in all transitions, but with varying degrees of clarity (Suppl. Fig. 3). The transition which looks most similar to the termination of the AHP is the transition between 380.2–377.2 kyr BP. The record begins with stable humid conditions, which are ended by a ~200 yr long moderately dry episode at 381.3–381.1 kyr BP, similar to the episode with high K values at 7.7 kyr BP, although its climatic origin is not clear (Fig. 1, Suppl. Fig. 3). A few hundred years later, the record shows a ~150 long extreme drought between 380.88–380.72 kyr BP, similar to the dry event at 7.7 kyr BP. These two extreme events are followed by a succession of wet and dry events, each lasting a few decades and recurring every ~100–200 years. Strikingly similar is the calming down of this flickering, i.e., the decrease of the amplitude in the time between ~380–377 years, similar to the decrease in the amplitudes of the flickering between 7–4 kyr BP. In both cases, at 380.2–377.2 kyr BP and 7–4 kyr BP, the Chew Bahir tentatively returned to wetter conditions–unlike other times, which were characterized by very rapidly recurring periods of moisture (Fig. 1, Suppl. Fig. 3, Suppl. Tab. 2).

The other examples also show many similarities, but also differences in the course of the decrease in humidity, for example, in the length of the transition, in the occurrence of the flickering, and also in details such as the decrease in the amplitude of the extreme events (Suppl. Fig. 3, Suppl. Tab. 2). On the one hand, this may indicate that the climate–or the Chew Bahir basin–does not always behave the same, the K proxy does not always record the same, or the proxy's record is disturbed by sedimentary processes. It would be fascinating to check whether it is similarities (or differences) in forcing that provoke repeated similar (or different) behavior in Chew Bahir. Unfortunately, the quality of the age model does not allow such a test: the accuracy of the age model is not better than 1–2 precession cycles[7,10]. Overall, however, the great similarity in the character of the recurrent transitions, including the pronounced flickering between extremely wet and dry conditions, probably indicates that this form of transition is a climatic change typical of the region.

## Supplementary references


1. Trauth, M. H. *et al*. Episodes of environmental stability versus instability in late cenozoic lake records of eastern Africa. *J. Hum. Evol.* **87**, 21–31 (2015).
2. Trauth, M. H. *et al*. Classifying past climate change in the Chew Bahir basin, southern Ethiopia, using recurrence quantification analysis. *Clim. Dyn.* **53**, 2557–2572 (2019).
3. Trauth, M. H. *et al*. Abrupt or gradual? change point analysis of the late Pleistocene–Holocene climate record from Chew Bahir, southern Ethiopia. *Quat. Res.* **90**, 321–330 (2018).
4. Foerster, V. *et al*. Climatic change recorded in the sediments of the Chew Bahir basin, southern Ethiopia, during the last 45,000 years. *Quat. Int.* **274**, 25–37 (2012).
5. Foerster, V. *et al*. Towards an understanding of climate proxy formation in the Chew Bahir basin, southern Ethiopian rift. *Palaeogeogr. Palaeoclimatol. Palaeoecol.* **501**, 111–123 (2018).
6. Davies, S.J. *et al*. Micro-XRF Core Scanning in Palaeolimnology: Recent Developments., in: Croudace, I.W., Rothwall, R.G., Micro-XRF Studies of Sediment Cores, Applications of a non-destructive Tool for the Environmental Sciences. *Developments in Paleoenvironmental Research* **17**, 189–226 (2015).
7. Foerster, V. *et al*. 620,000 years of eastern African climate variability and hominin evolution. *Nat. Geosci.* **15**, 805–811 (2022).
8. Trauth, M. H. *et al*. Recurring types of variability and transitions in the 620 kyr record of climate change from the Chew Bahir basin, southern Ethiopia. *Quat. Sci. Rev.* **266**, 106777 (2021).
9. Davidson, A. The Omo River Project: reconnaissance geology and geochemistry of parts of Ilubabor, Kefa, Gemu Gofa and Sidamo. Tech. Rep., Ethiopian Institute of Geological Surveys Bulletin (1983).
10. Roberts, H. M. *et al*. Using multiple chronometers to establish a long, directly-dated lacustrine record: Constraining ~600,000 years of environmental change at Chew Bahir, Ethiopia. *Quat. Sci. Rev.* **266**, 107025 (2021).
11. Reimer, P. J. *et al*. The IntCal20 northern hemisphere radiocarbon age calibration curve (0–55 cal kBP). *Radiocarbon* **62**, 725–757 (2020).

## Supplementary tables

**Suppl. Tab. 1** Cored locations within the Chew Bahir basin (longitudes and latitudes), core lengths, total depth of cored material below the lake floor, date of core collection, and core recovery.

| Core ID | Location | Latitude | Longitude | Length | Total Depth | Coring Date | Core Recovery |
|---|---|---|---|---|---|---|---|
| CB01 | Margin | N 04°50.6’ | E 36°46.8’ | 22 m | 19 m | Dec 2009 | 81% |
| CB02 | Margin | N 04°48.7’ | E 36°46.2’ | 10 m | 9 m | Nov 2010 | 97% |
| CB03 | Intermediate | N 04°47.9’ | E 36°47.2’ | 11 m | 11 m | Nov 2010 | 98% |
| CB04 | Centre | N 04°43.3’ | E 36°50.2’ | 10 m | 10 m | Nov 2010 | 99.5 % |
| CB05 | Centre | N 04°42.8’ | E 36°51.3’ | 10 m | 10 m | Nov 2010 | 97% |
| CB06 | Centre | N 04°44.1’ | E 36°47.9’ | 10 m | 10 m | Nov 2010 | 97% |
| **CB01–06** | | **Composite Core** | | | **19 m** | | **~95%** |
| | | | | | | | |
| **CHB14-1** | **Center** | **N 4°42.4'** | **E 36°51.1'** | | **~40 m** | | |
| | | | | | | | |
| CHB14-2A | Margin | N 4°45.7' | E 36°46.1' | | 278.58 m | Nov/Dec 2014 | |
| CHB14-2B | Margin | N 4°45.7' | E 36°46.2' | | 266.38 m | Nov/Dec 2014 | |
| **CHB14-2** | | **Composite Core** | | | **292.87 m** | | **~90%** |

**Suppl. Tab. 2** Comparison of selected wet-dry transitions in the Chew Bahir Record during the past 620 kyr BP. Duration describes the length of a ramp-shaped transition from wet to dry. Hereby, many of the transitions never reach stable dry conditions, but are followed by a rapid return to wet conditions. In this case, the beginning of this return is used as the end point of the ramp and thus for calculating the duration.

| | Transition (kyr BP) | Duration (kyr) | Appearance of flickering during transition |
|---|---|---|---|
| A | 4.3–7 | 2.7 | Very clear flickering in both directions, calming down towards the end. |
| B | 189.1–190.7 | 1.6 | Yes, very clear flickering after the tipping point, similar to AHP. |
| C | 211.7–215 | 3.8 | Yes, but relatively long (~150 yr) wet events, but short droughts. |
| D | 290–292 | 2 | Two ~150 yr long wet episodes, some shorter ones. |
| E | 309.3–314.4 | 5.1 | Multiple wet and dry excursions. |
| F | 377.2–380.2 | 3 | Flickering in both directions, decreasing towards the end, similar to AHP. |
| G | 457–465 | 8 | Flickering very similar to that at the end of the AHP. |
| H | 488–495.6 | 7.6 | Clear flickering but also two extreme wet excursions of unknown origin. |
| J | 519.3–535 | 15.7 | Very unstable climate during transition, strong flickering. |
| K | 592.7–598.7 | 6 | Increasing instability, different from 377.2–380.2 kyr transition. |

## Supplementary figures

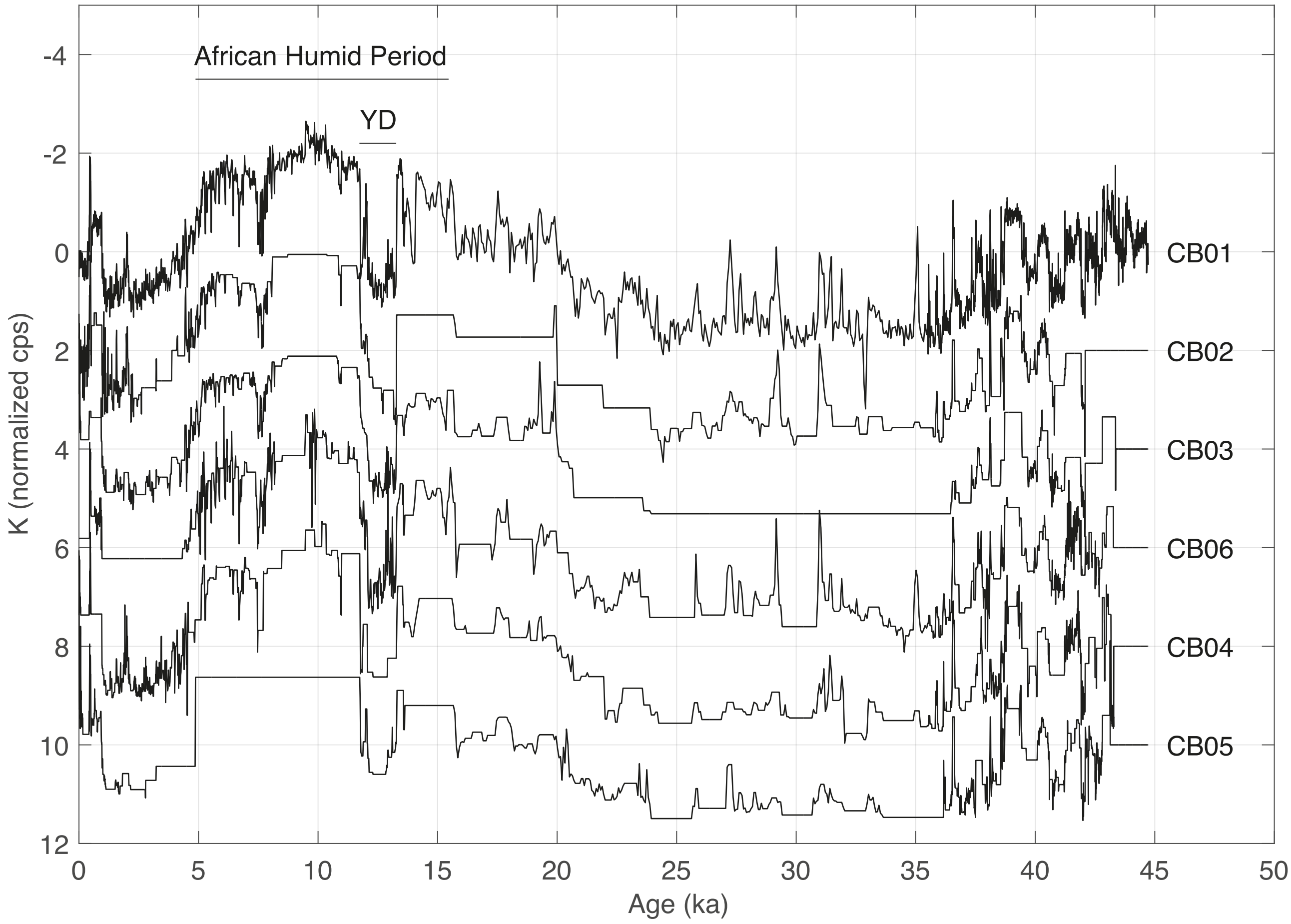


**Suppl. Fig. 1** Variation in the potassium (K) content (in standardized counts per second, cps) of the Chew Bahir cores CB01–06 plotted against age (in ka). We used dynamic time warping (DTW) to automatically align the K records from cores CB02 to CB06 with that from core CB01. The core depths of the aligned cores were converted into ages using the linear age model[1] with radiocarbon ages recalibrated using IntCal20[11]. Please note reverse scale of potassium axis. We have offset the individual time series by 2 standardized cps in each case to allow an overlap-free display of the K records.

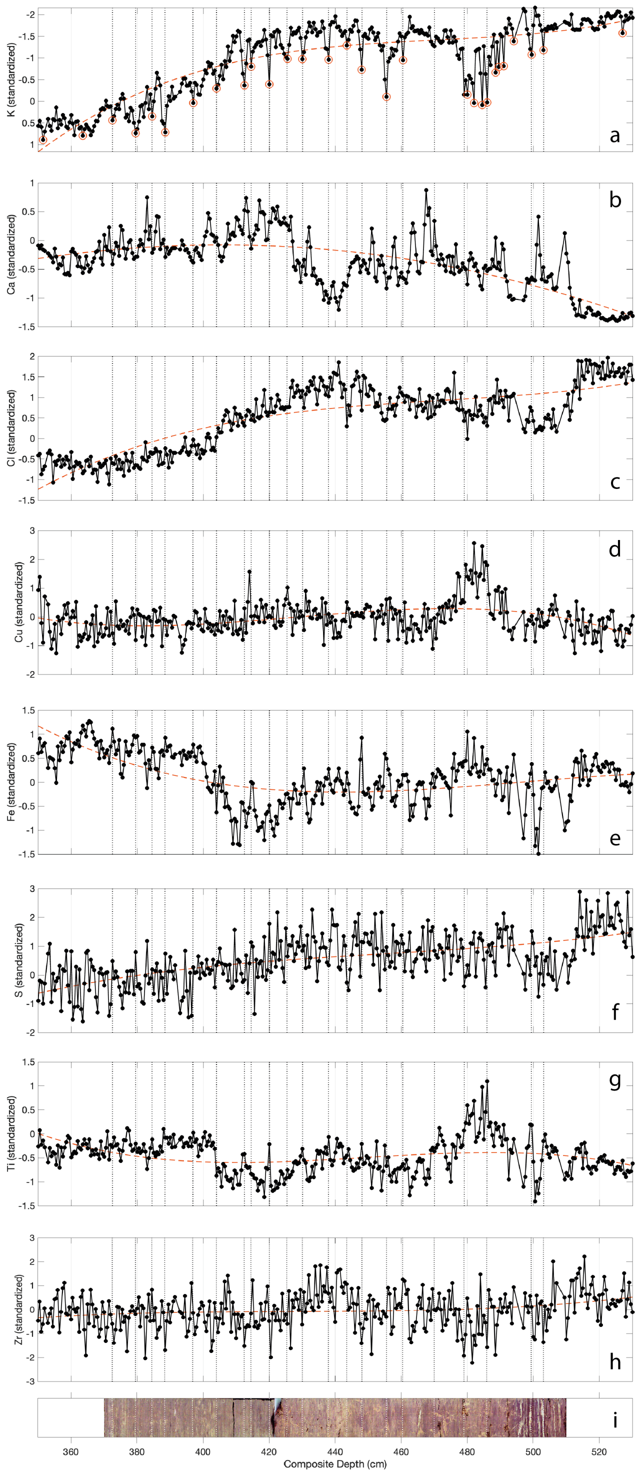


**Suppl. Fig. 2** Elemental concentrations of **a** potassium (K), **b** calcium (Ca), **c** chlorine, **d** copper (Cu), **e** iron (Fe), **f** sulfur (S), **g** titanium (Ti), and **h** zirconium (Zr) measured by µXRF scanning, and **i** core image in the range between ~370 and ~510 cm composite depth (bottom panel). Dotted vertical lines indicate the high K events, which are discussed as possible droughts and precursor events of the tipping point at the termination of the AHP.

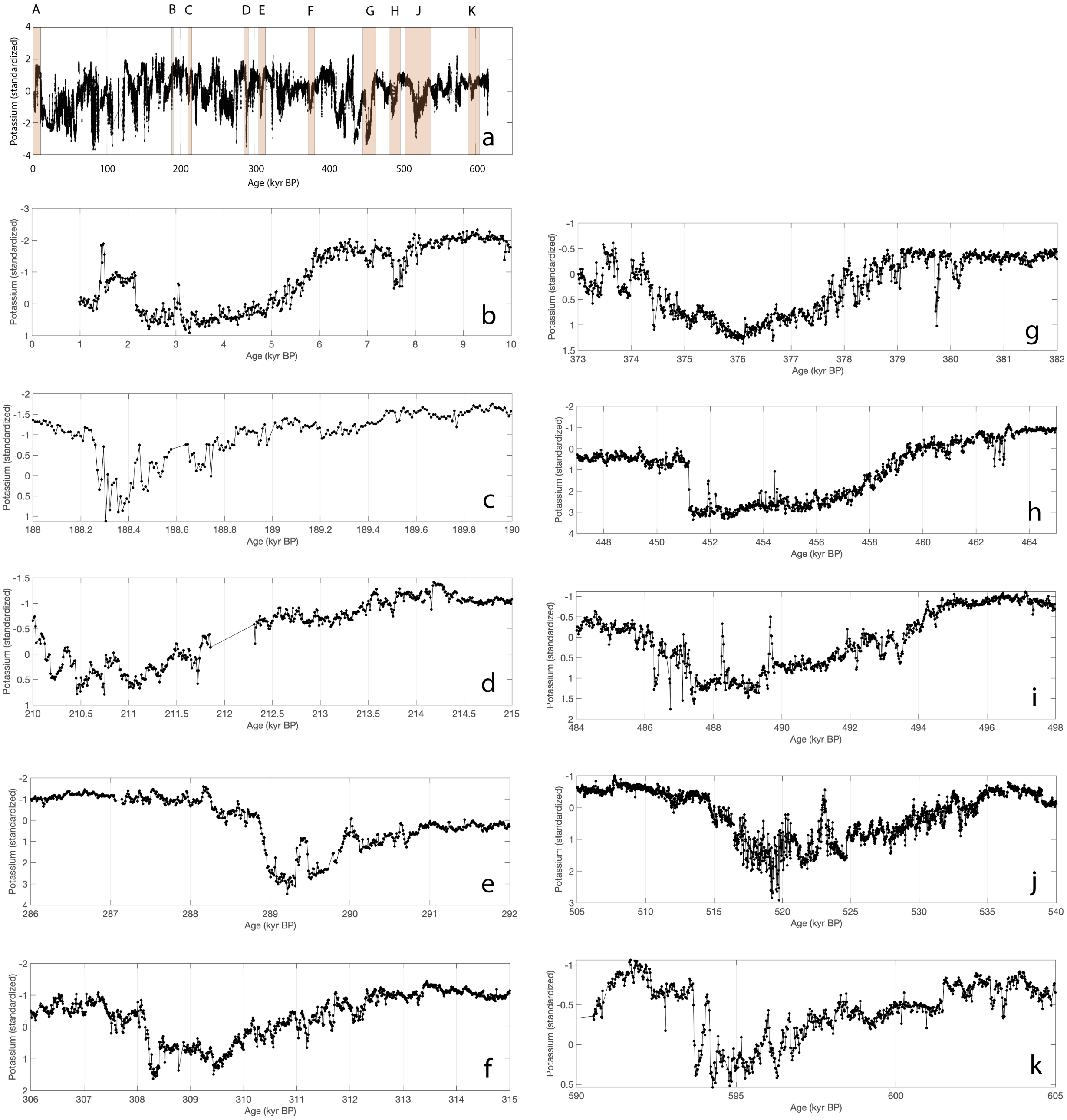


**Suppl. Fig. 3 a–k** Selection of possible wet-dry transitions/tipping points with precursors in the long core from the Chew Bahir basin from Suppl. Tab. 2. The transitions, although very similar on a long time scale, differ in the occurrence of flickering and early-warning signals.

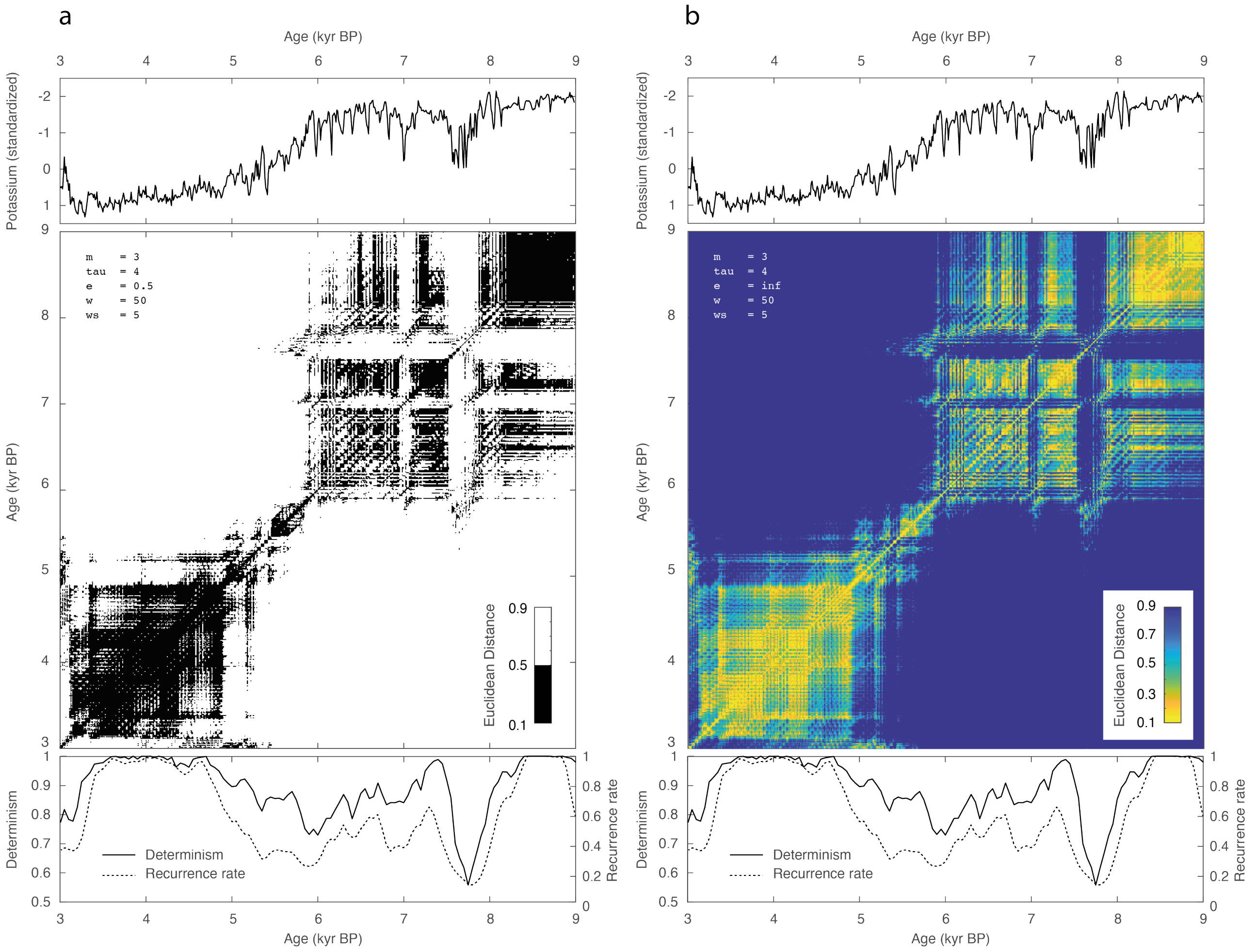


**Suppl. Fig. 4 a** Thresholded (*e*=0.5) (left panel) and **b** unthresholded (*e*=inf) (right panel) recurrence plot and recurrence quantification analysis of the record of relative aridity in the Chew Bahir basin during the 9–3 kyr BP interval. Time series with 10 yrs resolution after interpolation (upper panel), global or unthresholded recurrence plot (middle panel), and recurrence rate of a moving window (lower panel). Embedding dimension *m*=3, delay *tau*=4, window size for calculating the recurrence rate *w*=50 (corresponding to 500 yrs) and lag to move the window *ws*=5 (50 yrs). See the text for a detailed description of the embedding parameters measures, the interpretation of recurrence plots and the recurrence rate.

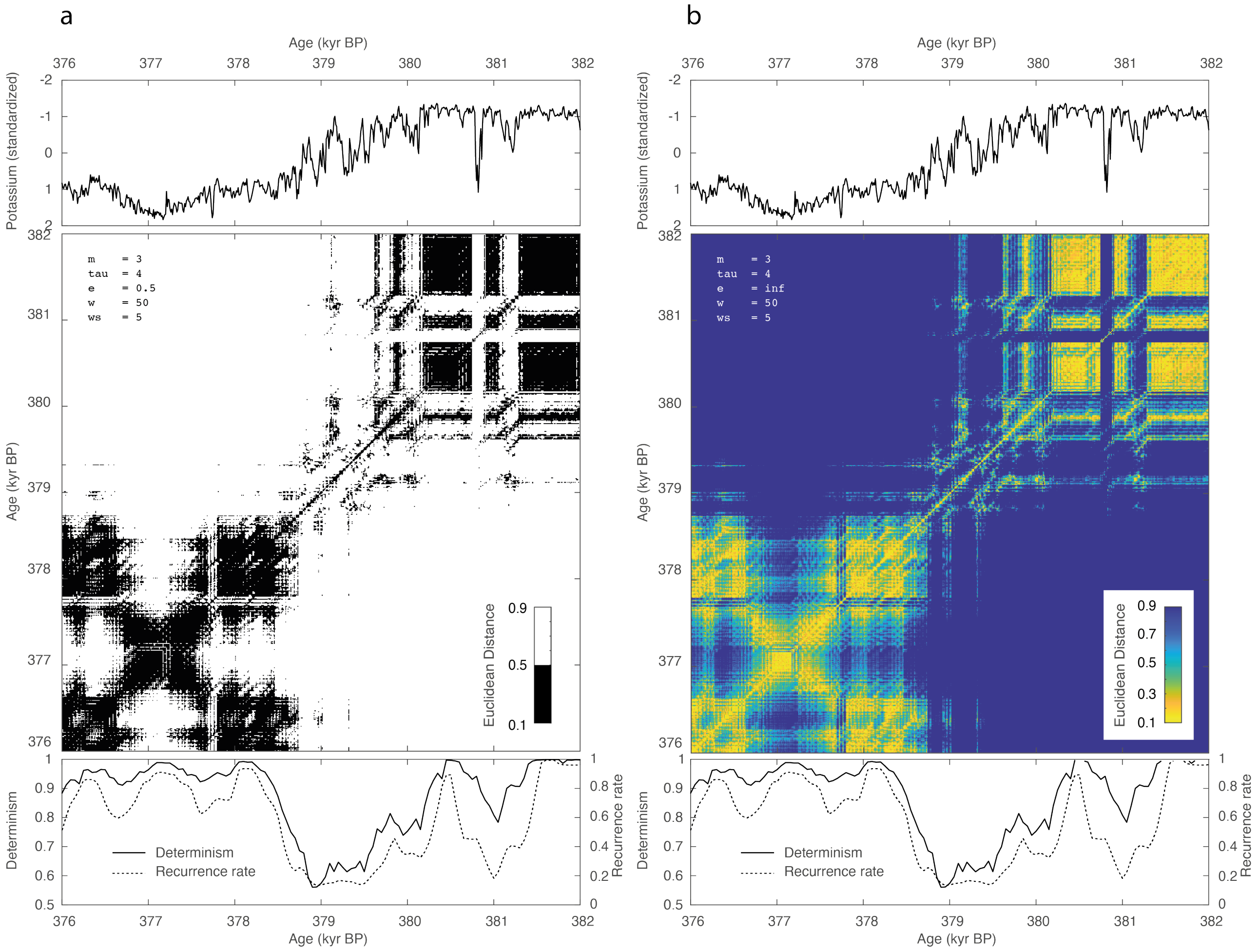


**Suppl. Fig. 5 a** Thresholded (*e*=0.5) (left panel) and **b** unthresholded (*e*=inf) (right panel) recurrence plot and recurrence quantification analysis of the record of relative aridity in the Chew Bahir basin during the 382–376 kyr BP interval. Time series with 10 yrs resolution after interpolation (upper panel), global or unthresholded recurrence plot (middle panel), and recurrence rate of a moving window (lower panel). Embedding dimension *m*=3, delay *tau*=4, window size for calculating the recurrence rate *w*=50 (corresponding to 500 yrs) and lag to move the window *ws*=5 (50 yrs). See the text for a detailed description of the embedding parameters measures, the interpretation of recurrence plots and the recurrence rate.

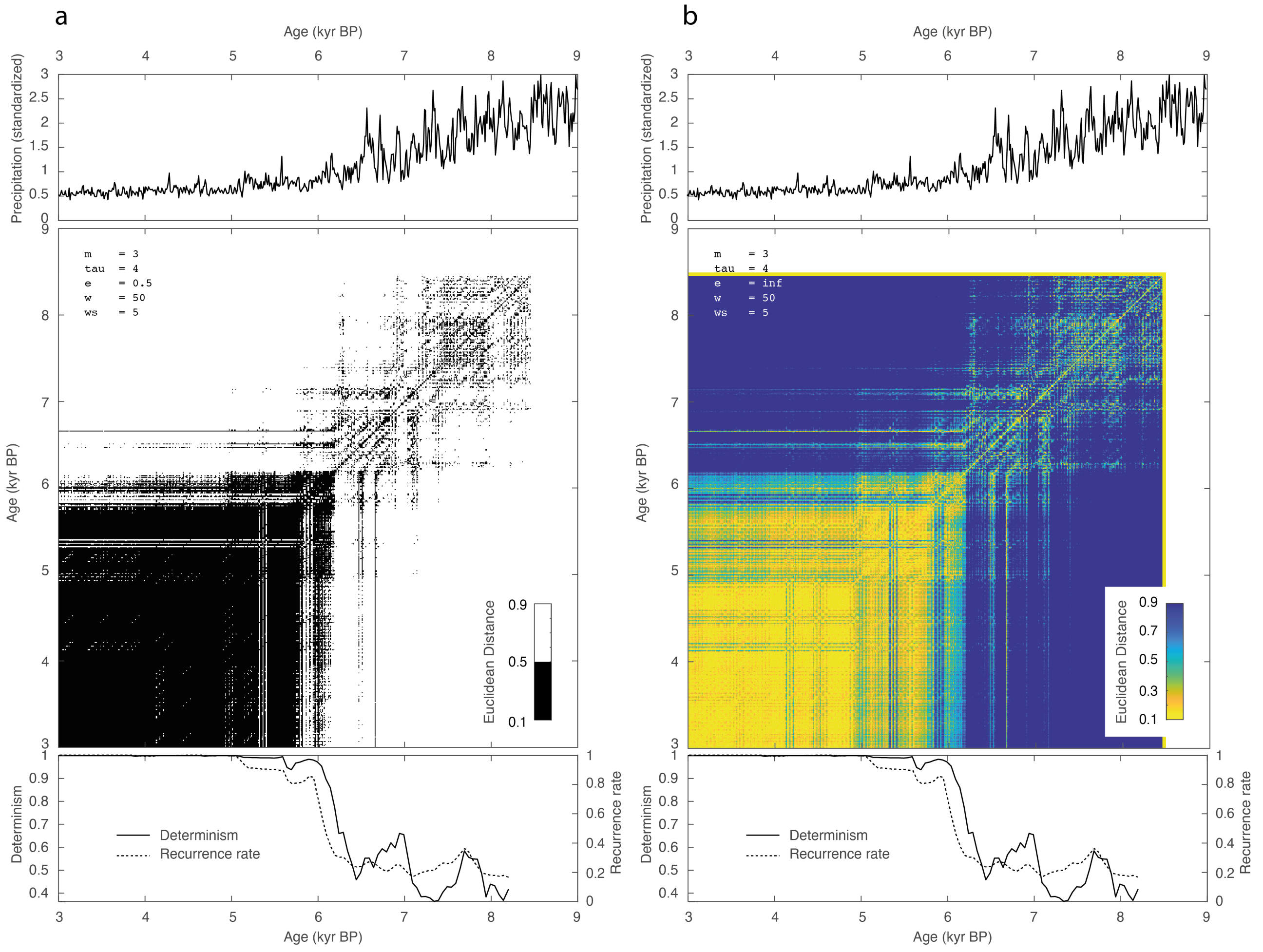


**Suppl. Fig. 6 a** Thresholded (*e*=0.5) (left panel) and **b** unthresholded (*e*=inf) (right panel) recurrence plot and recurrence quantification analysis of modeled precipitation (averaged over 20–30°N by 20°W–5°E for June–July–August) during the 9–3 kyr BP interval. Time series with 10 yrs resolution after interpolation (upper panel), global or unthresholded recurrence plot (middle panel), and recurrence rate of a moving window (lower panel). Embedding dimension *m*=3, delay *tau*=4, window size for calculating the recurrence rate *w*=50 (corresponding to 500 yrs) and lag to move the window *ws*=5 (50 yrs). See the text for a detailed description of the embedding parameters measures, the interpretation of recurrence plots and the recurrence rate.

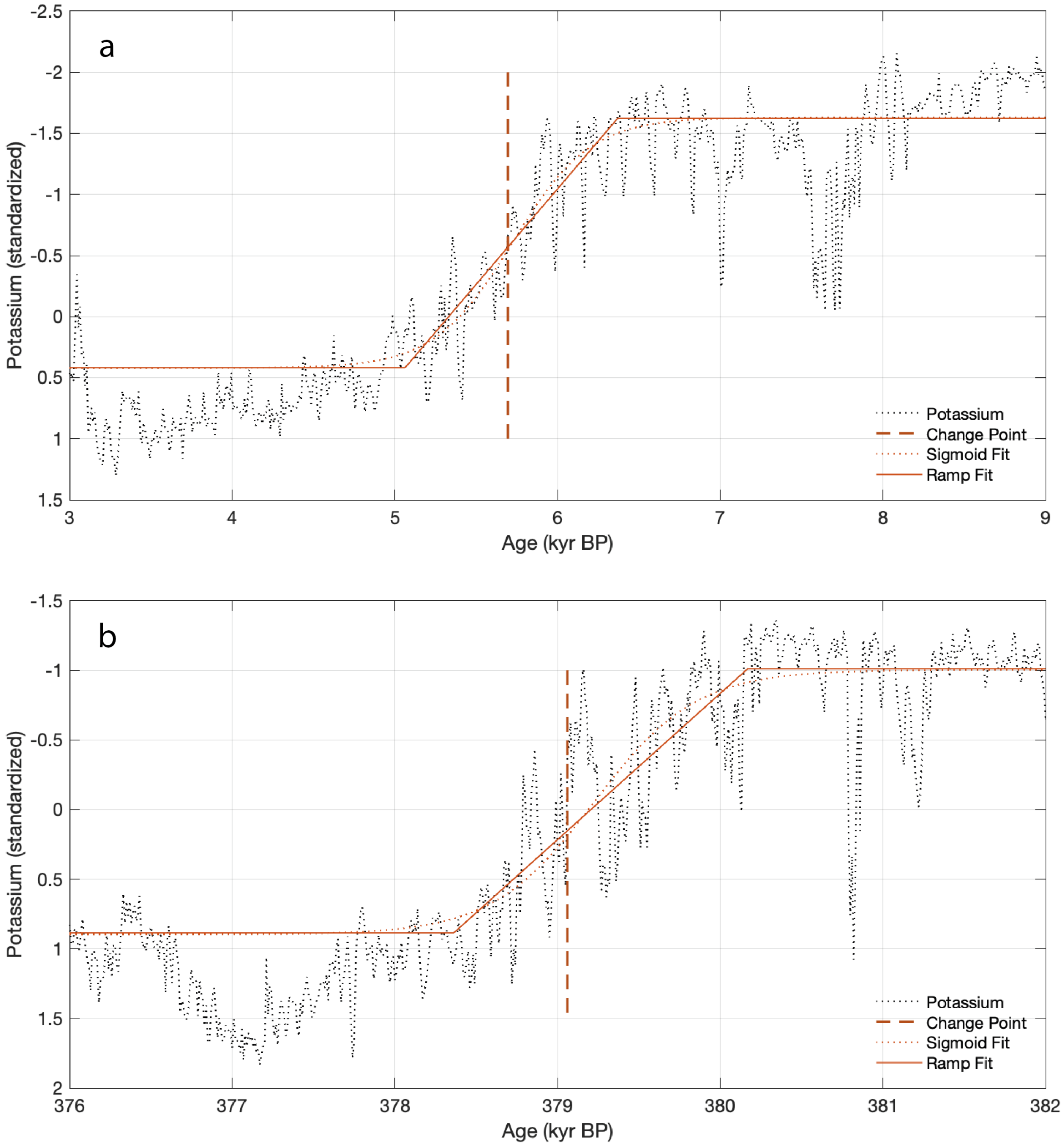


**Suppl. Fig. 7** Results of a change point analysis (dashed red line) and nonlinear least-squares fitting of a sigmoid function (dotted red line) and a ramp function (solid red line) to the **a** 9–3 kyr BP interval and the **b** 382–376 kyr BP interval of the K curve from Chew Bahir (dotted black line).
